# Secretory autophagy promotes Rab37-mediated exocytosis of tissue inhibitor of metalloproteinase 1

**DOI:** 10.1186/s12929-022-00886-z

**Published:** 2022-12-02

**Authors:** Shan-Ying Wu, Jia-Wen Chen, Hsi-Yu Liu, Yi-Ching Wang, Yeh-Shiu Chu, Chi-Ying Huang, Kai-Ying Lan, Hsiao-Sheng Liu, Sheng-Hui Lan

**Affiliations:** 1 Department of Microbiology and Immunology, School of Medicine, College of Medicine, Taipei Medical University, Taipei, Taiwan; 2 Graduate Institute of Medical Sciences, College of Medicine, Taipei Medical University, Taipei, Taiwan; 3 Department of Microbiology and Immunology, College of Medicine, National Cheng Kung University, Tainan, Taiwan; 4 Department of Pharmacology, College of Medicine, National Cheng Kung University, Tainan, Taiwan; 5 Institute of Basic Medical Sciences, College of Medicine, National Cheng Kung University, Tainan, Taiwan; 6 Brain Research Center, National Yang Ming Chiao Tung University, Taipei, Taiwan; 7 Institute of Biopharmaceutical Sciences, National Yang Ming Chiao Tung University, Taipei, Taiwan; 8 M.Sc. Program in Tropical Medicine, College of Medicine, Kaohsiung Medical University, Kaohsiung, Taiwan; 9 Center for Cancer Research, College of Medicine, Kaohsiung Medical University, No. 100, Shin-Chuan 1st Road, Sanmin Dist., Kaohsiung City, 80708 Taiwan; 10 Department of Life Sciences and Institute of Genome Sciences, National Yang Ming Chiao Tung University, No. 155, Sec. 2, Linong Street, Taipei, 112 Taiwan (R.O.C.); 11 Cancer Progression Research Center, National Yang Ming Chiao Tung University, Taipei, Taiwan

**Keywords:** Autophagy, Rab37, TIMP1, Lung cancer, Lung-to-lung metastasis model

## Abstract

**Background:**

Rab37-mediated exocytosis of tissue inhibitor of metalloproteinase 1 (TIMP1), an inflammatory cytokine, under serum-depleted conditions which leads to suppression of lung cancer cell metastasis has been reported. Starvation is also a stimulus of autophagic activity. Herein, we reveal that starvation activates Rab37 and induces autophagy.

**Methods:**

We used an overexpression/knockdown system to determine the relationship between autophagy and Rab37 in vitro and in vivo. The autophagy activity was detected by immunoblotting, transmission electron microscope, autophagosome purification, and immunofluorescence under the confocal microscope. Lung-to-lung metastasis mouse model was used to clarify the role of autophagy and Rab37 in lung cancer. Clinical lung cancer patient specimens and an online big database were analyzed.

**Results:**

Initially, we demonstrated that active-form Rab37 increased LC3-II protein level (the marker of autophagosome) and TIMP1 secretion. Accordingly, silencing of Rab37 gene expression alleviated Rab37 and LC3-II levels as well as TIMP1 secretion, and induction of autophagy could not increase TIMP1 exocytosis under such conditions. Moreover, silencing the *Atg5* or *Atg7* gene of lung cancer cells harboring active-mutant Rab37 (Q89L) led to decreased autophagy activity and TIMP1 secretion. In the lung-to-lung metastasis mouse model, increased TIMP1 expression accompanied by amiodarone-induced autophagy led to decreased tumor nodules and cancer cell metastasis. These phenomena were reversed by silencing the *Atg5* or *Atg7* gene. Notably, increasing autophagy activity alone showed no effect on TIMP1 secretion under either Rab37 or Sec22b silencing conditions. We further detected colocalization of LC3 with either Rab37 or TIMP1, identified Rab37 and Sec22b proteins in the purified autophagosomes of the lung cancer cells harboring the active-form Rab37 gene, and confirmed that these proteins are involved in the secretion of TIMP1. We reveal that autophagic activity was significantly lower in the tumors compared to the non-tumor parts and was associated with the overall lung cancer patient survival rate.

**Conclusions:**

We are the first to report that autophagy plays a promoting role in TIMP1 secretion and metastasis in a Rab37-dependent manner in lung cancer cells and the lung-to-lung mouse model.

**Supplementary Information:**

The online version contains supplementary material available at 10.1186/s12929-022-00886-z.

## Background

Lung cancer is one of the leading cancers to cause death worldwide [1]. Non-small-cell lung cancer (NSCLC) and small-cell lung cancer (SCLC) are the most common types. Approximately 85% of patients belong to NSCLC, which consists of three subtypes: lung adenocarcinoma (LUAD), lung squamous cell carcinoma (LUSC), and lung large cell carcinoma [2, 3]. Over 70% of lung cancers proceed to advanced metastasis in brain, bone, adrenal gland, and liver by the interaction between cancer cell surface proteins and endothelial-cell receptors at distant sites [4]. Therefore, the demand for preventing the malignant development of NSCLC is urgent.

Autophagy maintains homeostasis through selective or nonselective recruitment of cargos (aggregated protein, damaged organelle, pathogen and microRNA) in the cytosol followed by fusion with the lysosome for degradation to eliminate stress-caused damage [5–8]. Moreover, a novel function designated secretory autophagy is attracting more attention from scientists. Unlike degradative autophagy, the recruited cytosolic cargoes in the autophagosome are transported to the extracellular environment (exocytosis) [9]. Secretory autophagy machinery participating in the exocytosis of cytokines (IL-1β, IL-6, IL-18 and TNF-α), aggregated proteins, organelle, and microbial secretion has been reported [9, 10]. Bustos et al. reported that secretory autophagy is involved in intercellular communication of the tumor microenvironment (TME) through released cargo [11]; however, what molecules and functions are involved and the underlying mechanisms remain elusive.

Rab proteins, a small GTPase, function as vesicle trafficking including endocytosis, vesicle transition, and exocytosis [12]. Rab proteins regulate the initiation and termination of vesicle trafficking by cycling between GDP bound inactive Rab and GTP bound active Rab [13]. Functionally, Rab8, Rab11, Rab21, Rab23, Rab25 and Rab27B promote cancer cell migration and tumor invasion [14–20]. In contrast, Rab17, Rab25 and Rab37 suppress tumorigenesis [21]. Moreover, some Rab members are known to be involved in the canonical degradative autophagy machinery including Rab8b [12, 22]. However, Rab8a is responsible for the secretion of unconventional proteins through secretory autophagy [23–25]. Tsai et al. reported that under starvation conditions, Rab37-anchored vesicles secrete the tissue inhibitor of metalloproteinase 1 (TIMP1) to inactivate matrix metalloproteinase 9 (MMP9) and suppress cancer cells migration both in vitro and in vivo [26]. Notably, Schroeder et al. demonstrated that starvation activated Rab7 (active-form Rab7) leads to the fusion of the lipid droplet with the autophagosome named lipophagy [27]. Moreover, we reveal that high glucose activates both Rab37 and autophagy, and Rab37/autophagy, in combination promotes insulin secretion in vitro and in vivo [28]. Altogether, the above knowledge indicates that Rab proteins need to be activated and then execute their functions in the cell.

In this study, we clarified whether starvation could activate Rab37 and induces autophagy simultaneously in lung cancer cells. We further validated the role of Rab37-autophagy-mediated TIMP1 secretion in tumorigenesis of lung cancer both in vitro and in vivo together with clinical lung cancer patient specimens.

## Materials and methods

### Cell lines and reagents

The lung cancer cell lines H460 control and H460-shRab37 knockdown cells were established from parental H460 cells and the stable lung cancer cell lines expressing active-form Rab37 (Q89L) and vector control transgenes were established from parental CL1-5 cells by Dr. Yi-Ching Wang (National Cheng Kung University, Tainan, Taiwan). The stable cell lines CL1-5-Rab37-Q89L-shGFP, shAtg5, shAtg7, and shSec22b cells were established by infection with lentivirus containing shRNAs (Academia Sinica, Taipei, Taiwan) followed by selection with puromycin (Sigma, MO, USA). All cells were grown in Dulbecco’s modified Eagle’s medium (DMEM, Gibco, NY, USA) with 10% fetal bovine serum (Gibco) and penicillin/streptomycin (Sigma) at 37 °C in a 5% CO_2_ incubator. Amiodarone, niclosamide, and spautin-1 were purchased from Sigma, MO, USA).

### GTP-agarose pulldown assay

Cells were treated under different conditions and followed by protein extraction. Protein lysate was incubated with ActivX Desthiobiotin-GTP probe™ (Thermo Scientific, IL, USA) overnight at 4 °C. The beads were centrifuged and washed twice with GTP-binding buffer [50 mM Hepes (pH 7.4), 1% Triton X-100, 10% glycerol, 150 mM NaCl, 1.5 mM MgCl_2_, 1 mM EGTA, and 1% protease inhibitor cocktail]. The GTP-bound proteins were resuspended with SDS gel loading buffer and the GTP-Rab37 protein expression was detected using anti-Rab37 antibody by immunoblotting.

### Immunofluorescent staining

The cells were seeded onto a 2-well chamber slide (SPL, Korea) and treated with different conditions. Anti-TIMP1 (ab109125, Abcam), anti-LC3 (PM036, M186-3, MBL), or anti-Rab37 (LTK BioLaboratories, Taiwan) antibody was used. The fluorescent change of the cells was detected under a multi-photon confocal microscope (Olympus, FV1000MPE, Tokyo, Japan). A detailed description is provided in our previous report [29].

### Immunoblotting

The total protein of the cell lysates was extracted after treatment with different conditions. A detailed description of the preparation is presented in our previous report [29]. The primary antibodies used to detect specific proteins were β-actin (A544, Sigma), LC3 (PM036, Medical and Biological Laboratories, Nagoya, Japan), TIMP1 (ab109125, Abcam, Cambridge, UK), Rab37 (LTK BioLaboratories, Taiwan), ATG5 (ab108327, Abcam), and ATG7 (ab133528, Abcam). Samples were incubated overnight at 4 °C. The membranes were incubated with the secondary anti-rabbit (Amersham Pharmacia, Piscataway, NJ, USA) or anti-mouse (Chemicon, Temecula, CA, USA) antibody at room temperature for 1 h. Finally, the membrane was rinsed with enhanced chemiluminescence (ECL) (WBKLS0500; Millipore) and exposed using the BioSpectrum AC system (101-206-009; UVP, Upland, CA, USA).

### Immunoprecipitation

The extracted protein (1 mg) and beads were incubated with LC3 or IgG antibody. This mixture was rolled at 4 °C overnight. Beads were collected by centrifugation at 10,000 rpm for 1 min at 4 °C followed by extensively washing with PBS buffer and resuspending with SDS gel loading buffer. The protein samples were analyzed by immunoblotting.

### Preparation of conditional medium (CM)

Cells were cultured in a 6 cm dish with 4 ml serum-free medium for 24 h to prepare the conditional medium. The media were collected using Amicon Ultra centrifugal filter units (UFC900396, Millipore) according to the manufacturer’s protocol, and the total protein concentration in conditional medium samples was determined by Bradford assay (Bio-Rad Laboratories Inc.).

### Transmission electron microscopy (TEM)

The cell and autophagosome fraction (AP) were fixed with 2.5% glutaraldehyde in 0.1 M cacodylate buffer at 4 °C for 10 min. A detailed account of the procedure for sample preparation is described in a previously published study [29]. The samples were then sectioned, and the sliced samples were investigated under a TEM (Hitachi, Japan, HITACHI-7000).

### Negative staining of the purified autophagosome by immunogold labeling

In brief, 4 μl of purified autophagosome was loaded onto the carbon film supported nickel grid (CF400 Ni, EMS Microscopy Academy). After 1 min, the sample was removed by Whatman filter paper. LC3 protein on the surface of the autophagosome was recognized by the primary antibody (M152-3, MBL), which was diluted 50-fold in HB buffer. After 1 h, 10-nm gold immunogold-conjugated antibody (Abcam), which was diluted 50-fold in HB buffer, was used to detect the primary antibody. Finally, the grid was washed once by ddH_2_O, and the sample was negatively stained with 2% uranyl acetate for 1 min. The samples were investigated under a TEM (JEOL, USA, JEM-1400) at 120 kV with a Gatan Model 895 digital camera.

### Total internal reflection fluorescence (TIRF) imaging

Cells received the same treatment as immunofluorescent staining. A TIRF system was equipped with a high-sensitivity EMCCD Camera (iXOn3897, Andor technology) and an UPON 100XOTIRF objective lens (NA = 1.49; Olympus). We tracked each vesicle trafficking distance with track IT software (Olympus) and a thickness of 100 μm.

### Autophagosome purification

Cells (in 10% sucrose) were mixed with 0.5 ml of the buffer (1 M Hepes/0.1 M EDTA) and homogenized using a Dounce homogenizer. This homogenate was diluted with homogenization buffer (HB; 0.25 M sucrose, 10 mM HEPES, 1 mM EDTA, pH 7.3) containing 1.5 mM glycyl-l-phenylalanine 2-naphthylamide (GPN) and 1% DMSO, to a final GPN concentration of 0.5 mM and a final homogenate concentration of 5%. After incubation for 7 min at 37 °C to destroy the lysosomes, the homogenate was cooled to 4 °C. The tubes of GPN-treated homogenate were centrifuged at 4000 revolutions per minute (rpm) for 2 min to collect the supernatant, which was placed on top of a discontinuous (two-step) Nycodenz gradient, prepared by diluting isotonic (36% w/v) Nycodenz with HB to obtain a top layer of 9.5% Nycodenz and a bottom layer of 22.5% Nycodenz. The Nycodenz gradient was centrifuged overnight at 26,000 rpm in an SW28 rotor (Beckman, USA), and the gradients were divided into three fractions. The interface band was diluted with HB and layered on top of a discontinuous gradient of 33% Percoll in HB on top of 22.5% Nycodenz in HB, and centrifuged for 1 h at 20,000 rpm in the SW28 rotor. Autophagosomes formed a band at the lower interface, from which it could be recovered for analysis. Percoll was removed from the fractions of interest (e.g., the autophagosomes) by mixing with isotonic 60% (w/v) iodixanol in water, and centrifuging for 30 min in an SW40 rotor at 20,000 rpm. The autophagosome band was collected from the interface band [29].

### In solution digestion and LC–MS/MS analyses

The protein was extracted from the autophagosomes of CL1-5 cells. Protein extracts were denatured and then alkylated in dithiothreitol (7 mM) and iodoacetamine (21 mM), respectively, at 37 °C for 30 min. Proteins were digested by trypsin at 37 °C for 16 h. The peptide mixtures were separated on a 3 × 150 mm C18 column (Gemini, Phenomenex, Torrance, CA, USA) coupled to a high-performance liquid chromatography system (Beckman Coulter, CA, USA) using an acetonitrile gradient in 0.1% ammonium hydroxide solution, with about 30 fractions. The peptide fractions were analyzed on a nanoLC-Q Exactive™ HF mass spectrometer (Thermo Fisher, San Jose, USA) equipped with an HPLC system (M Class, Waters, MA, USA). MS raw files were uploaded into Proteome Discoverer (version 2.1, Thermo Fisher, MA, USA) with the default setting to generate peak lists for protein identification using the MASCOT search engine (version 2.5, Matrix Science, MA, USA) against the Swiss-Prot Mus musculus protein database (released in Jan 2016). The peptides sharing an identical sequence among multiple proteins were assigned to the one with the highest protein score. The identification of peptides and proteins with a false discovery rate of less than 1% was considered acceptable.

### Lung-to-lung metastasis assay

Male NOD/SCID mice (6–8 weeks old) were obtained from the Laboratory Animal Center of National Cheng Kung University (Tainan, Taiwan). The experimental protocol complied with Taiwan’s Animal Protection Act and was approved by the Laboratory Animal Care and Use Committee of National Cheng Kung University. A total of 5 × 10^4^ cells were suspended in 100 ml matrigel (BD Biosciences) in serum-free DMEM. Mice were anesthetized by intraperitoneal administration of Zoletil 50 solution (10 mg/ml, Virbac, Taipei, Taiwan) combined with 2.3 mg/ml of Xylazine Hydrochloride (also named Rompun) (Bayer Leverkusen, Germany) at a dose of 0.8 to 1 mg/10 g of body weight. After mice had been anesthetized with Zoletil 50 solution, they were placed in the left lateral decubitus position. Tuberculin syringes (10 μl) with 23-gauge hypodermic needles were used to inject the cell inoculum percutaneously into the right lateral thorax, at the lateral dorsal axillary line, 1.5 cm above the lower rib line, just below the inferior border of the scapula. The needle was quickly advanced 5–7 mm into the thorax and was slowly removed after the injection of cell suspension. After the tumor injection, the mouse was turned to the right lateral decubitus position. Animals were observed for 45–60 min until fully recovered.

### Immunohistochemistry (IHC)

The slides were prepared as described in our recent report [29]. The primary antibody was used to detect TIMP1 (ab109125, Abcam). The Dako REAL Envision Detection system (Dako, Glostrup. Denmark) was used to detect the protein expression. Hematoxylin (Merk, Darmstadt. Germany) was used to demonstrate nuclear morphology, and DAB (Dako) was used in conjugation with immunoperoxidase detection systems.

### Statistical analysis

Data are presented as the mean ± standard deviation (SD) values (error bars). Differences between the experimental and control groups were analyzed by two-tailed Student’s *t*-test. P value was considered statistically significant: *p < 0.05, **p < 0.01, and ***p < 0.001.

## Results

### Starvation induces the activities of Rab37 and autophagy together with increased TIMP1 secretion

**Fig. 1:**
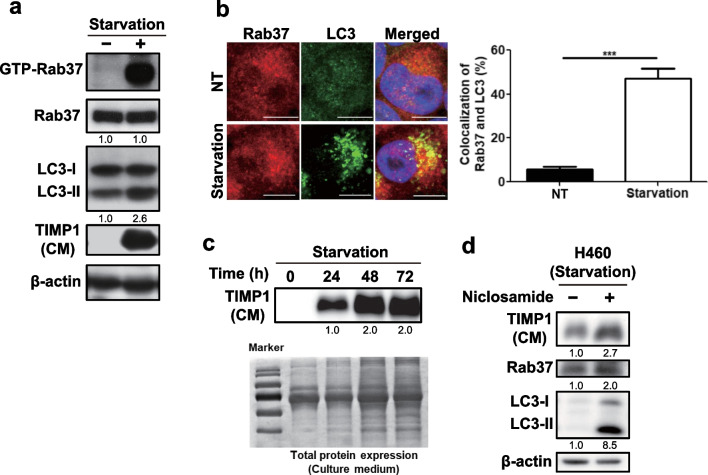
Starvation induces the activities of Rab37 and autophagy together with increased TIMP1 secretion. **a** Lung cancer CL1-5 cells were treated with serum-free medium for 24 h. The level of TIMP1 protein in the conditional medium (CM), as well as the levels of active-form and total Rab37, LC3, and β-actin in cell lysate, were investigated using specific antibodies by immunoblotting. Active-form Rab37 (GTP-Rab37) was pulled down by GTP-binding beads in total cell lysate followed by Rab37 antibody detection. **b** Under starvation for 24 h, colocalization of Rab37 (red) and LC3 (green) labeled by secondary antibody conjugated rhodamine and FITC, respectively were investigated under multi-photon confocal microscopy. Scale bar = 10 μm. The quantification of colocalization of Rab37 and LC3 was conducted by randomly counting 30 cells. **c** The levels of secreted TIMP1 in CM of lung cancer H460 cells were evaluated under starvation conditions for 0, 24, 48, and 72 h by immunoblotting. Total proteins in the gel stained by Coomassie blue dye were used to represent equal loading. **d** Lung cancer cell H460 was treated with 0.5 μM niclosamide for 24 h under starvation conditions. The level of secreted TIMP1 (CM) as well as the levels of total Rab37, LC3, and β-actin in cell lysate, were measured by immunoblotting

It has been reported that under serum-depleted conditions, Rab37-mediated exocytosis of TIMP1 suppresses MMP9, which leads to decreased metastases of lung cancer cells in vivo [26]. Starvation (serum-depleted conditions) is also a well-known inducer of autophagy. Herein, we reveal that serum starvation not only increased the level of active-form Ras37 accompanied by increased TIMP1 secretion but also increased the level of LC3-II in lung cancer CL1-5 cells (Fig. 1a). We then clarified whether autophagy participates in active-form Rab37-mediated TIMP1 secretion in lung cancer cells. Our image investigation revealed increased colocalization (yellow) of Rab37 (red) and LC3 (green, representing autophagosome) under starvation conditions (Fig. 1b). We further showed that starvation increased TIMP1 secretion in a time-dependent manner (Fig. 1c). Balgi et al*.* screened 3584 chemicals for mTORC1 inhibitors (also autophagy inducers). They identified four potent FDA-approved candidates including niclosamide and amiodarone [30]. Amiodarone and niclosamide can induce autophagic activity and autophagic flux has been reported [8, 28]. In this study, both amiodarone and niclosamide were used. Under starvation conditions, TIMP1 secretion in condition medium (CM) was further boosted when the H460 lung cancer cells were treated with niclosamide (Fig. 1d). These findings imply that starvation induced activities of both Rab37 and autophagy, which are involved in TIMP1 secretion.

### Autophagy promotes TIMP1 secretion in a Rab37-dependent manner under starvation conditions

**Fig. 2:**
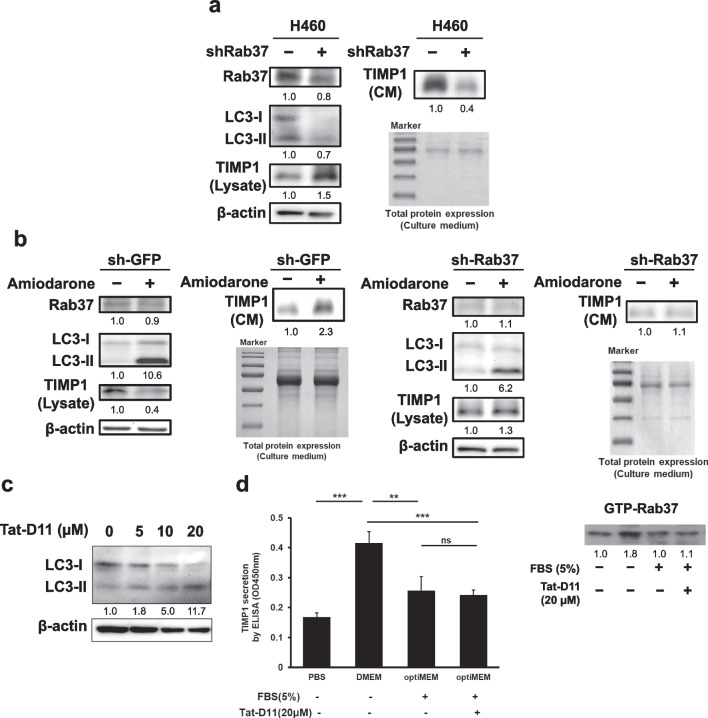
Autophagy promotes TIMP1 secretion in a Rab37-dependent manner under starvation conditions. Lung cancer H460 cell line stably expressing Rab37 lentivirus shRNA was established. **a** Total protein of the H460 cells was extracted, and the protein levels of Rab37, LC3, and TIMP1 in cell lysate and in condition medium (CM) were collected for detection of specific cellular proteins as well as the secreted TIMP1 by immunoblotting using specific antibodies. β-Actin and the whole cell lysate with Coomassie blue staining were used as the internal controls. **b** H460 cells stably expressing lentivirus Rab37 or GFP shRNA were treated with amiodarone (2 μM) for 24 h. The protein level of TIMP1 in the conditional medium (CM), and the levels of Rab37, TIMP1, and LC3 protein in the cell lysate were evaluated by immunoblotting using specific antibodies. **c** OptiMEM is similar to DMEM and is specially used for Tat-D11 treatment. H460 cells were treated with optiMEM with 5% serum and different concentrations of Tat-D11 (0–20 μM) for 7 h, and the levels of LC3 protein were determined by immunoblotting using anti-LC3 antibody. β-Actin was used as the internal control. **d** H460 cells were treated with PBS, DMEM without 5% serum, optiMEM with 5% serum, and optiMEM + 5% serum + Tat-D11 (20 µM) for 7 h, and then condition media were collected, followed by ELISA detection to measure the level of TIMP1. In addition, active-form Rab37 (GTP-Rab37) in the total cell lysate of the four groups was pulled down by GTP-binding beads, followed by Rab37 antibody detection. **p < 0.01, ***p < 0.001, ns: not significant. Data were analyzed by Student’s *t*-test

To clarify the role of Rab37 in autophagy and TIMP1 secretion, Rab37 gene expression was silenced by lentiviral shRNA, and the levels of Rab37, LC3-II in lung cancer H460 cells, as well as the secretion of TIMP1 in condition media were decreased (Fig. 2a). Our data are consistent with the findings reported by Tsai et al. showing that Rab37 plays an essential role in TIMP1 secretion. Furthermore, when Rab37 gene expression was silenced (sh-Rab37) in H460 cells, amiodarone remained to be able to increase LC3-II level but had no effect on TIMP1 secretion compared to the shGFP control cells under serum starvation conditions (Fig. 2b). We further used Tet-D11™, a small peptide autophagy inducer, under normal conditions without activation of Rab37 to confirm the effect of autophagy alone on TIMP1 secretion. Our data showed that Tet-D11 increased LC3-II level in a dose-dependent manner under normal conditions (Fig. 2c). Accordingly, a high level of secreted TIMP1 was only detected in the cells under serum-depleted conditions [Fig. 2d, column 2, induced by starvation-induced active Rab37 (GTP-Rab37)]. In contrast, TIMP1 secretion was not induced in the cells with medium supplemented with 5% serum, regardless of with or without Tet-D11 treatment (Fig. 2d, column 1 vs. 3 and 4). This data confirmed that just induction of autophagy without active Rab37 could not increase TIMP1 secretion. These results indicated that starvation increased the activities of both Rab37 and autophagy. The former plays an essential role in TIMP1 secretion, and the latter mainly plays a promotive role in TIMP1 exocytosis.

### Active-form Rab37-mediated TIMP1 secretion correlates with autophagic activity and colocalization of Rab37 and LC3

**Fig. 3:**
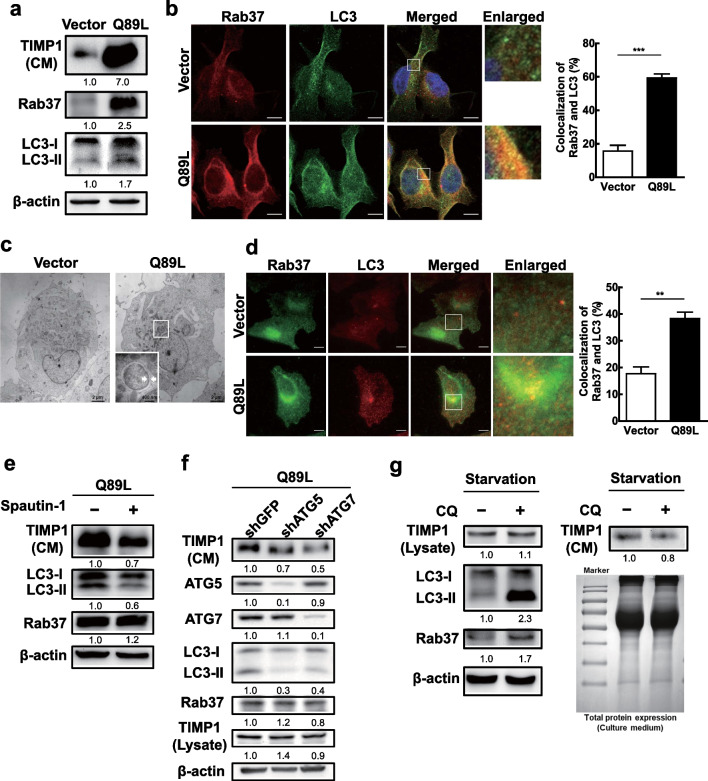
Active-form Rab37-mediated TIMP1 secretion was associated with increased autophagic activity and colocalization of Rab37 and LC3. **a** Lung cancer CL1-5 cells stably expressing active-form Rab37 (Q89L) or (Vector) plasmid were cultured for 24 h. The levels of TIMP1 in the conditional medium and the proteins of active-form Rab37, total Rab37, LC3, and β-actin in the cell lysate were evaluated by immunoblotting using specific antibodies. β-Actin was used as the internal control. **b** CL1-5 Vector and Rab37 Q89L cells were maintained in the serum-free medium for 24 h. Rab37 protein was labeled by primary anti-hRab37 antibody and second antibody conjugated with rhodamine (red). LC3 protein was labeled by primary anti-LC3 antibody and second antibody conjugated with FITC (green). The labeled cells were observed under confocal microscopy. The quantification of colocalization of Rab37 and LC3 was conducted by randomly investigating 30 cells. **c** TEM was used to investigate the double membrane autophagosome-like vesicles in Vector and O89L cells. The white arrow points to the double membrane autophagosome-like vesicle. **d** CL1-5-Vector and CL1-5-Q89L cells were seeded on the slides for 24 h. The same procedure as in **b** was used to label Rab37 protein (green) and LC3 protein (red) and investigated under a total internal reflection fluorescence (TIRF) microscope. Scale bar = 10 μm. The quantification of colocalization of Rab37 and LC3 was conducted by randomly investigating 30 cells. **e** CL1-5-Q89L cells were treated with autophagy inhibitor spautin-1 (10 μM) for 24 h. The protein level of TIMP1 in the conditional medium, and LC3 and Rab37 proteins in the total cell lysate were evaluated by immunoblotting. **f** ATG5 and ATG7 genes were silenced by specific lentivirus shRNAs in CL1-5-Q89L cells. Lentiviral shGFP was used as the negative control. The protein levels of Atg5, ATG7, LC3, Rab 37, and TIMP1 in cell lysate, as well as TIMP1 in the conditional medium were evaluated by immunoblotting. **g** CL1-5-Q89L cells were treated without serum in the presence or absence of CQ (50 μM) for 24 h. The levels of TIMP1 in the conditional medium, total LC3, TIMP1, Rab 37, and β-actin in the cell lysate were evaluated by immunoblotting using specific antibodies. β-Actin was used as the internal control

Based on our findings, we hypothesize that secretory autophagy participates in active-form Rab37-mediated vesicle trafficking of TIMP1. Initially, we compared the levels of TIMP1 secretion and LC3-II expression (representing autophagic activity) using CL1-5 lung cancer cell lines harboring either stably expressed active-form Rab37 (Q89L) or the control (Vector). We reveal that the level of active-form Rab37 (Q89L) correlated with increased TIMP1 secretion (CM) and LC3-II expression (Fig. 3a). Moreover, we observed increased LC3 punctum formation (representing autophagosomes) as well as colocalization of Rab37 and LC3 protein in active-form Rab37 (Q89L) cells compared to the control cells (Vector) under confocal microscopy (Fig. 3b). Similarly, we observed the double membrane autophagosome-like vesicles increased in active-form Rab37 (Q89L) cells compared to the control cells (Vector) under transmission electron microscopy (TEM) (Fig. 3c). Furthermore, increased colocalization of Rab37 (red) and LC3 (green) protein in proximity to the plasma membrane of active-form Rab37 (Q89L) cells was detected under a total internal reflection fluorescence microscope (TIRFM) (Fig. 3d). TIRFM is specifically used to investigate molecules that are less than 100 nm from the cellular membrane. Furthermore, TIMP1 secretion was decreased in the presence of active-form Rab37 (Q89L) when autophagy was suppressed by either autophagy inhibitor (spautin-1) (Fig. 3e) or genetic silencing of *ATG5* or *ATG7* gene using lenti-shRNAs (Fig. 3f). We further used an autophagy inhibitor chloroquine (CQ), a fusion blocker of autophagosome and lysosome, to block the degradative autophagy. The increased LC3-II accumulation in the presence of CQ in lung cancer cells indicates degradative autophagy was suppressed after CQ treatment. Importantly, both intracellular TIMP1 expression or TIMP1 secretion was not affected by CQ treatment (Fig. 3g). Our data indicate that serum-free starvation-induced degradative autophagic progression does not affect the level of intracellular and extracellular TIMP1 expression. In summary, active-form Rab37 (generated either by starvation or by expressing Rab37-Q89L mutant) mediated TIMP1 secretion associated with increased autophagic activity as well as colocalization of these two protein-anchored vesicles. Moreover, we discovered that only under active-form Rab37 status, the autophagy inducer increased TIMP1 secretion, and vice versa, i.e., the autophagy inhibitor decreased TIMP1 release. This finding implies that secretory autophagy plays a promotive role in TIMP1 secretion in an active-form Rab37-dependent manner.

### Ultrastructure of the autophagosome and recruited proteins

**Fig. 4:**
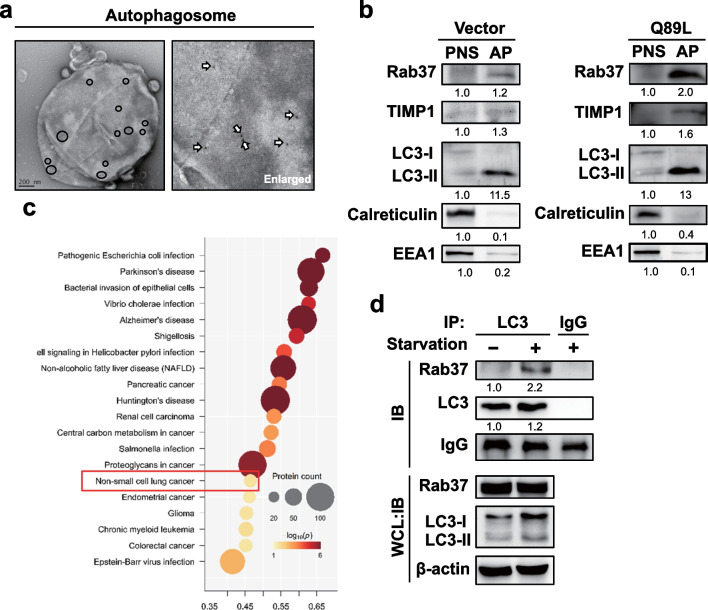
Ultrastructure of the autophagosome and recruited proteins. **a** After density gradient centrifugation, purified autophagosomes from the CL1-5-Q89L cells were collected. The LC3 protein in purified autophagosomes was labeled by anti-LC3 antibody conjugated with immunogold (12 nm) followed by negative staining and investigation under TEM. The circle and the white arrow indicate the location of the immunogold-labeled LC3 protein on the outside of the autophagosome membrane. **b** After centrifugation, the whole cell lysate of CL1-5-Q89L and -Vector cells were divided into: (1) the fraction of the post-nuclear supernatant (PNS); (2) the fraction of purified autophagosomes (AP). The levels of TIMP1, Rab37, LC3, EEA1 (endosomal marker), and calreticulin (endoplasmic reticulum marker) in PNS and AP fractions were evaluated using specific antibodies by immunoblotting. **c** Proteins in the purified autophagosome of CL1-5-Q89L cells were determined by LC–MS/MS, followed by KEGG database analysis to identify possible diseases. **d** Lung cancer CL1-5 cells were treated with serum-free medium for 24 h, and total protein extraction was immunoprecipitated by anti-LC3 antibody. The expression levels of proteins in the precipitates and whole cell lysates were evaluated by immunoblotting using specific antibodies. *WCL* whole cell lysate

We purified the autophagosome from CL1-5 lung cancer cells followed by immunogold-conjugated anti-LC3 antibody to label the LC3 protein. The specimens without section were treated with negative staining and investigated under an electron microscope. We found abundant immunogold-labeled LC3 protein on the outer membrane of the purified autophagosome by negative staining under TEM (Fig. 4a, circle and white arrow). We further purified autophagosomes from CL1-5 cells harboring active-form Rab37 (Q89L) and the control (Vector) cells under starvation conditions. The successful purification of autophagosomes was confirmed by high LC3 II expression and low background of calreticulin (endoplasmic reticulum marker) in the purified autophagosome (AP) compared to the post-nucleus supernatant (PNS) (Fig. 4b). To clarify whether Rab37 was involved in the endo-lysosomal trafficking pathway in our study, we analyzed the endosomal marker (EEA1) in purified autophagosomes under starvation conditions in lung cancer cells. We revealed a very small amount of EEA1 protein in the AP fraction compared to the PNS fraction from Vector and Q89L cells (Fig. 4b), indicating that endosomal trafficking was not involved in the Rab37-mediated secretory autophagy pathway.

We further detected increased levels of Rab37, TIMP1, and LC3-II in purified AP in CL1-5-Q89L cells (Fig. 4b), indicating that active-form Rab37 enhanced TIMP1 protein recruited into the autophagosomes. These proteins were also detected in the purified autophagosomes from lung cancer H460 cells under starvation conditions (Additional file 1: Fig. S1). We purified autophagosomes from the CL1-5-Q89L cells followed by Mass Spectrometry (MS)-based quantitative proteomics analysis. Our data reveal that most of the secretory autophagy-related proteins were detected in purified autophagosomes under the condition of active-form Rab37-mediated secretion of TIMP1 (Table 1). In addition, 26 proteins involved in the tumorigenesis of non-small cell lung cancer by KEGG analysis were identified in purified autophagosomes of CL1-5-Q89L cells (Fig. 4c and Table 2). These data imply that active-form Rab37-anchored vesicles may participate in the trafficking of proteins to the autophagosomes, followed by exocytosis with the help of secretory autophagy. Our recent publication indicated that Rab37 interacts with LC3 through LIR (LC3-interacting region) motif during glucose-stimulated insulin secretion in pancreatic β-cells [28]. Herein, we demonstrated that the interaction between Rab37 and LC3 was increased in lung cancer cells under starvation conditions by immunoprecipitation analysis (Fig. 4d, lane 2), which leads to the promotion of TIMP1 exocytosis by the secretory autophagy. Altogether, the trafficking of LC3-anchored vesicles was through a Rab37-dependent secretion process under starvation conditions.

**Table 1:** Rab37-mediated secretory autophagy related proteins identified from the autophagosome of CL1-5-Q89L

CL1-5-Q89L
LAMP1
LAMP2
MAP1LC3B2
RAB37
SEC22B
SFRP1
SNAP29
SQSTM1
TIMP1

**Table 2:** Protein related non-small cell lung cancer identified from autophagosome of CL1-5-Q89L

CL1-5-Q89L		
AKT1	GRB2	PIK3R1
AKT2	HRAS	PIK3R2
AKT3	KRAS	PLCG1
ARAF	MAP2K1	PLCG2
BRAF	MAP2K2	PRKCA
CDK4	MAPK1	RAF1
CDK6	MAPK3	SOS1
EGFR	NRAS	STK4
ERBB2	PDPK1	

^a^ All the identified proteins related to non-small cell lung cancer were analyzed by KEGG

### Amiodarone-induced autophagy promotes Rab37-mediated TIMP1 secretion and suppresses lung cancer cell metastasis in the lung-to-lung mouse metastasis model

**Fig. 5:**
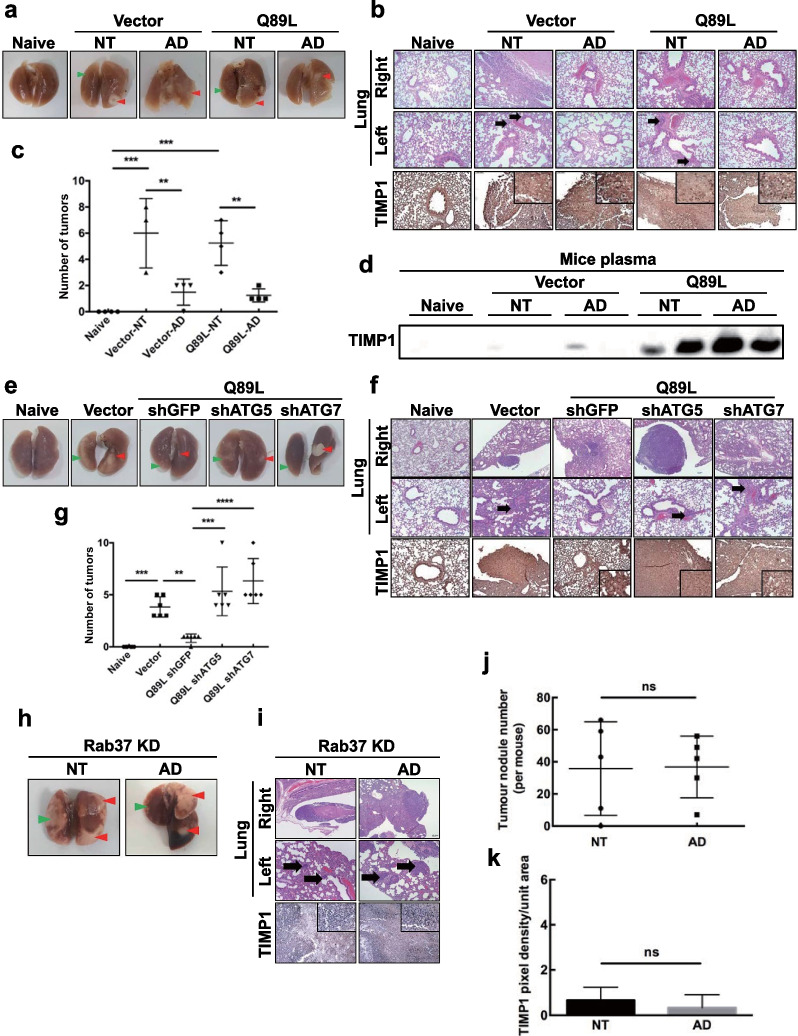
Autophagic promotes Rab37-mediated TIMP1 secretion and suppresses lung cancer cell metastasis in the lung-to-lung mouse metastasis model. Lung cancer CL1-5 control (Vector) and CL1-5-Q89L (Q89K) cells (5 × 10^4^ cells) were suspended in 10 μl of Matrigel. The Vector-AD and Q89L-AD group NOD-SCID mice received amiodarone (30 mg/kg) by i.p. injection twice a week for three weeks, together with the Naïve control group were sacrificed on day 35. Solid nodules located in the right lateral lung and within the pleural thoraxes were removed. **a** Five representative images of lungs were from the mice receiving CL1-5 Vector or CL1-5-Q89L cells with or without amiodarone together with the Naïve mice. Red arrow: primary tumor in the right lung; Green arrow: metastatic nodule in the left lung. **b** Row 1 and 2: H&E staining of lung tissues at local and distant regions of five groups. Row 3: IHC staining of the level of TIMP1 in the right lung tissues using anti-TIMP1 antibody. Scale bar = 10 μm. **c** Quantification of the lung tumors in the five groups of mice. **d** The five groups of NOD-SCID mice were inoculated i.p. with a single dose of amiodarone (30 mg/kg). Plasma samples were collected on the day of sacrifice, and TIMP1 protein level was measured by immunoblotting analysis using anti-TIMP1 antibody. Lung cancer Vector, CL1-5-Q89L-shGFP, -shAtg5, -shAtg7, and Naïve cells (5 × 10^4^ cells) suspended in 10 μl of Matrigel were inoculated into the five groups of mice (five mice/group). The solid nodules located in the right lung and within the pleural thoraxes were removed. **e** Five representative images of lungs were from the mice of the Naïve group and the mice receiving CL1-5-Q89L cells expressing Vector, -shGFP, -shAtg5, and -shAtg7 genes. Red arrow: primary tumor in the right lung; Green arrow: metastatic nodule in the left lung. **f** H&E stain showing tumor formation at the primary site (Row 1) and the distant regions (Row 2) in groups of Vector, Q89L-shAtg5, and Q89L-shAtg7 compared to the Q89L-shGFP group. Row 3: IHC stain showing the level of TIMP1 in the right lung tissues using anti-TIMP1 antibody. **g** Quantification of tumors in each group of mice. The NOD-SCID mice were divided into two groups (five mice/group). Lung cancer H460-shRab37 stable cells (5 × 10^4^ cells) suspended in 10 μl of Matrigel were inoculated into the right lung of the mice. The Rab37KD-AD group received amiodarone (30 mg/kg) by i.p. injection twice a week for two weeks. Mice were then sacrificed on day 35. The nodules located in the injected right lung and within the pleural thoraxes were removed. **h** Two representative images of lung tissues were inoculated with H460-shRab37 cells with (AD) or without amiodarone (NT). Red arrow: primary tumor in the right lung; Green arrow: metastatic nodule in the left lung. **i** Row 1 and 2: H&E staining of lung tissues at local and distant regions of the mice inoculated with H460 Rab37KD cells with (AD) or without amiodarone (NT). Row 3: IHC stain showing the level of TIMP1 in the left lung tissues using anti-TIMP1 antibody. Scale bars = 10 μm. **j** Quantification of tumors in the two mice groups. **k** Quantification of TIMP1 level in the two mice groups

To clarify the promotive role of autophagy in Rab37-mediated exocytosis of TIMP1 and suppression of metastasis in vivo, the autophagy inducer amiodarone was used. The lung-to-lung metastasis mouse model was prepared using lung cancer CL1-5 naïve, cells (Vector), and CL1-5-Q89L cells harboring active-form Rab37 transgene (Q89L). Mice were divided into five groups (five mice per group). Cancer cells were percutaneously inoculated into the right lateral thorax, and the tumor nodules in the left lungs of all the mice were verified by H&E staining. The amiodarone treatment groups were denoted as Vector-AD and Q89L-AD. Amiodarone (30 mg/kg) was inoculated into the mice twice a week for three weeks. All the mice were sacrificed on day 37 after inoculation. Compared to the naïve group (Naïve), all of the CL1-5-drived cells formed tumors in the primary sites of the right lung tissues. Moreover, the tumors were also detected in the left lungs in both the Vector-NT and Q89L-NT groups. The tumors in the right lungs (primary tumor) were labeled by red arrows, and the tumors in the left lungs (metastatic tumors) were labeled by green arrows (Fig. 5a). H&E staining confirmed that the metastatic nodules in the left lungs were only detected in the Vector-NT and Q89L-NT groups without amiodarone treatment (Fig. 5b, black arrow). No metastatic nodules were seen in the left lung tissues of amiodarone-treated groups (Fig. 5b). Moreover, TIMP1 expression was increased in the right lung tissue of the amiodarone-treated groups (Fig. 5b, row 3 for Vector-AD and Q89L-AD). Figure 5c shows the total number of tumors in the lungs detected in five groups. Both Vector-NT and Q89L-NT groups showed a higher number of tumor nodules in the right and left lungs compared to the other groups (Fig. 5c). High levels of TIMP1 were detected in the plasma of two mice inoculated with Q89L cancer cells after amiodarone treatment (Fig. 5d). To further clarify the role of autophagy in active-form Rab37-mediated TIMP1 secretion and lung cancer metastasis in vivo, the autophagy-essential genes *ATG5* or *ATG7* were silenced by lentiviral shRNA. Four CL1-5-Q89L derivatives harboring shATG5, shATG7, shGFP, and vector genes (Q89L-shATG5, Q89L-shATG7, Q89L-shGFP, and Vector) cells were inoculated into the right lungs of the mice, and the metastasis status of the tumor was monitored for 29 days. Compared to the control group (Naïve), all of the CL1-5-derived cells formed tumors in the primary sites of the injected lung tissues (Fig. 5e). Increased level of TIMP1 accompanied by reduced tumor numbers in the Q89L-shGFP group was detected compared to the Vector, Q89L-shATG5, and Q89L-shATG7 groups (Fig. 5f, g). Accordingly, the metastasis activity of lung cancer cells (black arrows) and TIMP1 expression were reversed in the Q89L-shATG5 and Q89L-shATG7 groups. Furthermore, we clarified the role of Rab37 in autophagy-promoted TIMP1 exocytosis by knockdown of Rab37 expression in vivo. The lung-to-lung metastasis mice model was prepared using Rab37 knockdown lung cancer H460 cells (Rab37 KD H460). Mice were divided into two groups (five mice per group). The Rab37 KD-AD group received amiodarone by i.p. injection (30 mg/kg) twice a week for two weeks, followed by sacrifice and evaluation of tissues. The mice with or without amiodarone treatment formed metastasis tumors in the left lungs (Fig. 5h). Compared to the non-treatment group (NT), there was no significant difference in the number of metastatic tumor nodules in the left lung tissues of the amiodarone-treated group (black arrows) (Fig. 5i, j). In addition, there was no difference in TIMP1 expression in right lung tissue between the NT and AD groups under the Rab37 knockdown system in vivo (Fig. 5k), indicating that Rab37 is required for TIMP1 secretion. Our in vivo data consistent with in vitro data imply that secretory autophagy plays a promoting role in TIMP1 secretion in a Rab37-dependent manner, which leads to suppression of metastasis in vivo.

### The effects of Sec22b on Rab37 and autophagy-mediated TIMP1 secretion and tumorigenesis in lung cancer H460 cells.

Autophagy progression involves multiple membrane fusion steps, including endosome—multiple vesicle body- and lysosome-autophagosome fusion. SNAREs play an essential role in the membrane fusion of transport vesicles with an acceptor compartment. In mammalian cells, there are over 36 SNARE members that are localized in different membrane compartments. Sec22b, a SNARE family member, is involved in vesicle and membrane fusion in secretory autophagy progression [31, 32]. Sec22b-labelled autophagosomes are delivered to the membrane of another vesicle and interact with SNAP23/29 and Stx3/4 SNARE proteins anchored on the plasma membrane responsible for cytokine secretion [33]. Based on the above findings, the role of Sec22b in Rab37-autophagy-mediated TIMP1 secretion is noteworthy for exploration.

**Fig. 6:**
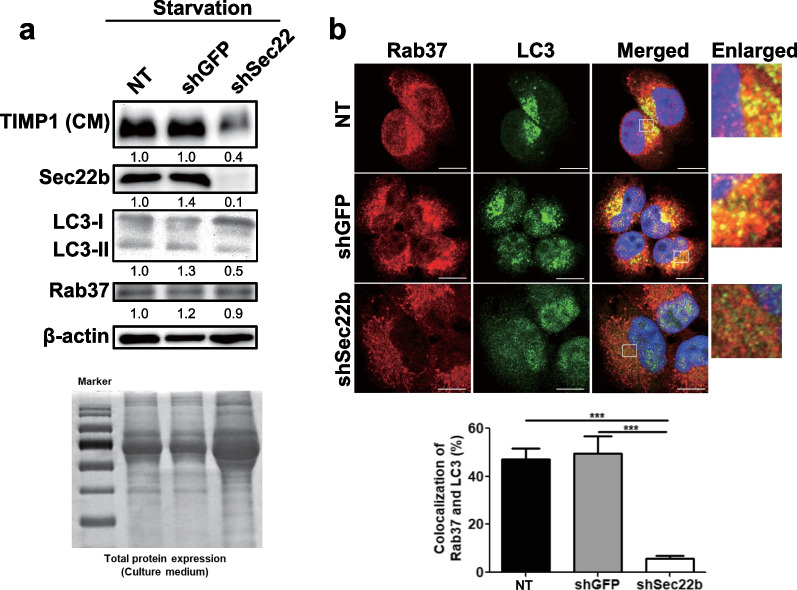
The effects of Sec22b on Rab37 and autophagy-mediated TIMP1 secretion and tumorigenesis in H460 cells. **a** The level of secreted TIMP1 in condition medium and the levels of Sec22b, LC3, and Rab37 in the cell lysate were determined by immunoblotting using specific antibodies. β-actin and Coomassie gel staining of the total proteins represent equal loading controls of the proteins in the cells and in the supernatant, respectively. **b** Colocalization of rhodamine-labeled Rab37 (red) and FITC-labeled LC3 (green) was investigated under starvation conditions for 24 h under a multi-photon confocal microscope. Quantification of colocalization of Rab37 and LC3 was conducted by randomly counting 30 cells. Scale bar = 10 µm. Error bars represent mean ± SD. Data were analyzed by Student’s t-test; ***p < 0.001

We clarified the role of Sec22b in Rab37 and autophagy-mediated TIMP1 secretion by lentiviral shRNA. Under starvation conditions, silencing of Sec22b resulted in decreased levels of Sec22b, LC3-II, and TIMP1 secretion compared to the control (NT) and shGFP H460 cells (Fig. 6a). Image analysis data confirmed that the number of LC3 puncta and Rab37-LC3 colocalization decreased in shSec22b cells compared to the NT and shGFP cells (Fig. 6b). Moreover, Rab37 protein level and distribution were not changed in shSec22b cells compared to control cells (Fig. 6a, b). In summary, Sec22b positively affects LC3-II level, LC3 puncta number, Rab37-LC3 colocalization, and TIMP1 secretion.

### Analysis of significance of p62 accumulation and Sec22b level using clinical lung cancer specimens and an online database

**Fig. 7:**
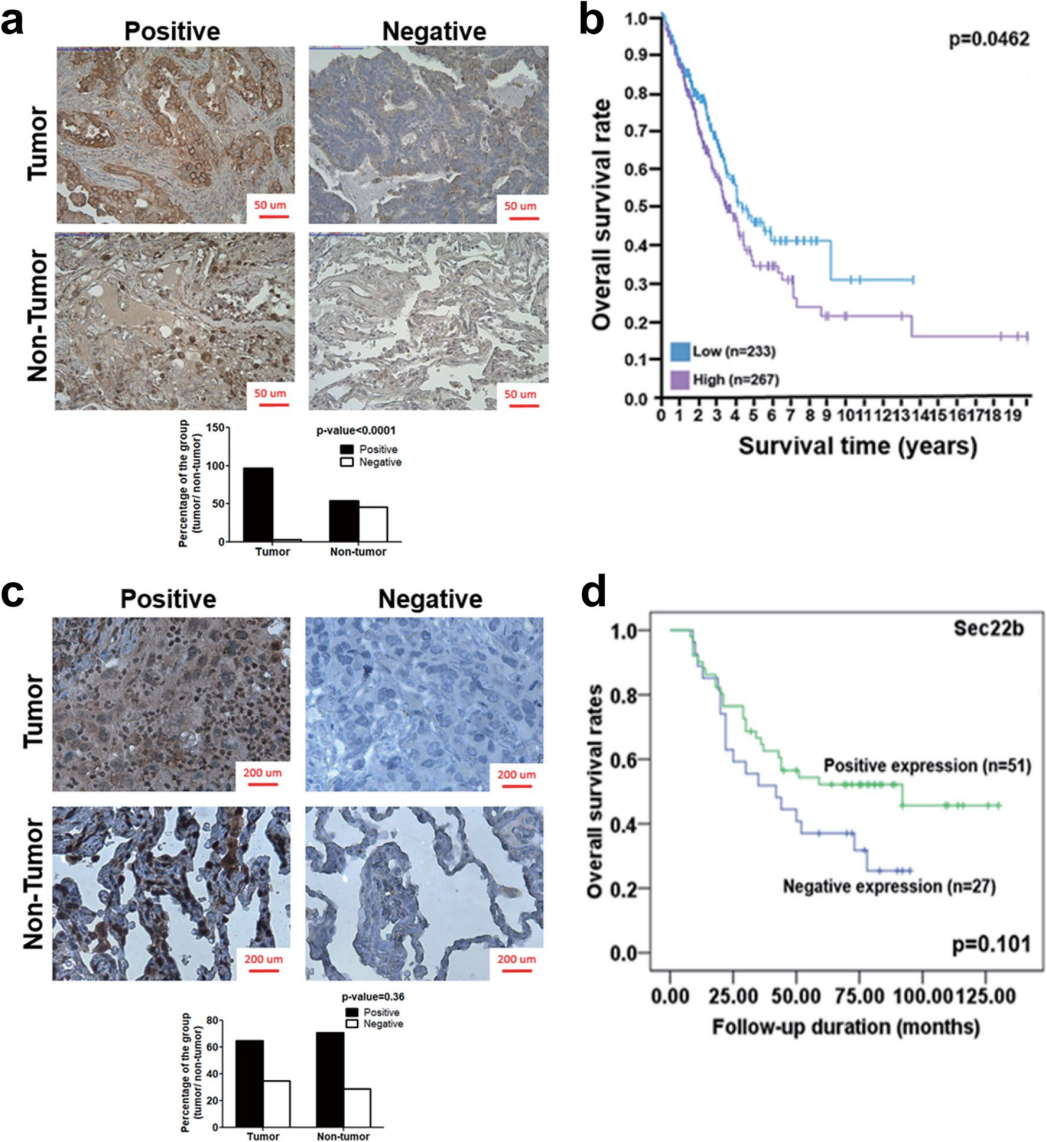
Analyses of significance of p62 accumulation and Sec22b level in clinical lung cancer specimens and an online database. **a** The sections of 103 lung cancer patient specimens were labeled by anti-p62 antibody followed by DAB staining (brown). Hematoxylin (blue) was used to stain the nuclei. Quantification of p62 level in lung cancer specimens is shown as a bar graph. Data were analyzed by chi-square test. Scale bar = 50 µm. **b** The survival rate of lung adenocarcinoma (LUAD) patients in an online database (n = 500) was calculated using Kaplan–Meier analysis and log-rank test (THE HUMAN PROTEIN ATLAS, https://www.proteinatlas.org/). **c** The sections of 136 lung cancer patient specimens were labeled by anti-Sec22b antibody. Quantification of Sec22b expression in lung cancer specimens is shown as a bar graph. Data were analyzed by chi-square test. Scale bar = 50 µm. **d** The survival rate of the lung cancer patients was determined by Kaplan–Meier survival analysis and log-rank test

Our findings imply that autophagy together with Sec22b plays a pivotal role in Rab37-mediated TIMP1 secretion and tumorigenesis of lung cancer cells. Herein, we clarified the effects of autophagy and Sec22b status by analyzing lung cancer patient specimens as well as an online TCGA big database. We analyzed four tissue arrays of lung cancer patient specimens and an online lung cancer patient database to evaluate p62 and Sec22b protein levels. The p62 protein, an autophagic substrate, is widely used as a marker of autophagy progression [34]. High p62 accumulation represents low autophagy degradation activity. Our data show that both the level of p62 accumulation and the percentage of high p62 accumulation specimens were high in tumor parts compared to the non-tumor parts. About 97% of the tumor tissue showed positive p62 accumulation. Only 54% of the non-tumor parts showed positive p62 accumulation (Fig. 7a, Tumor vs. Non-tumor). We further analyzed the online database of lung cancer patients (UCSC Xena), and the results showed that in adenocarcinoma lung cancer patients, a high accumulation of p62 protein correlated with a worse overall survival rate (Fig. 7b, *p* = 0.0462).

We also investigated the Sec22b protein level in the same set of lung cancer patient specimens. However, the protein level of Sec22b showed no difference between the tumor parts and normal parts. About 65% of the tumor tissue showed positive Sec22b expression, and 71% of the non-tumor parts showed positive Sec22b expression (Fig. 7c). We also found no correlation between the level of Sec22b and the overall survival rate of the lung cancer patients (Fig. 7d, *p* = 0.101). These clinical data imply that autophagic activity was significantly lower in the tumors compared to the non-tumor parts and was associated with the overall lung cancer patient survival rate.

## Discussion

**Fig. 8:**
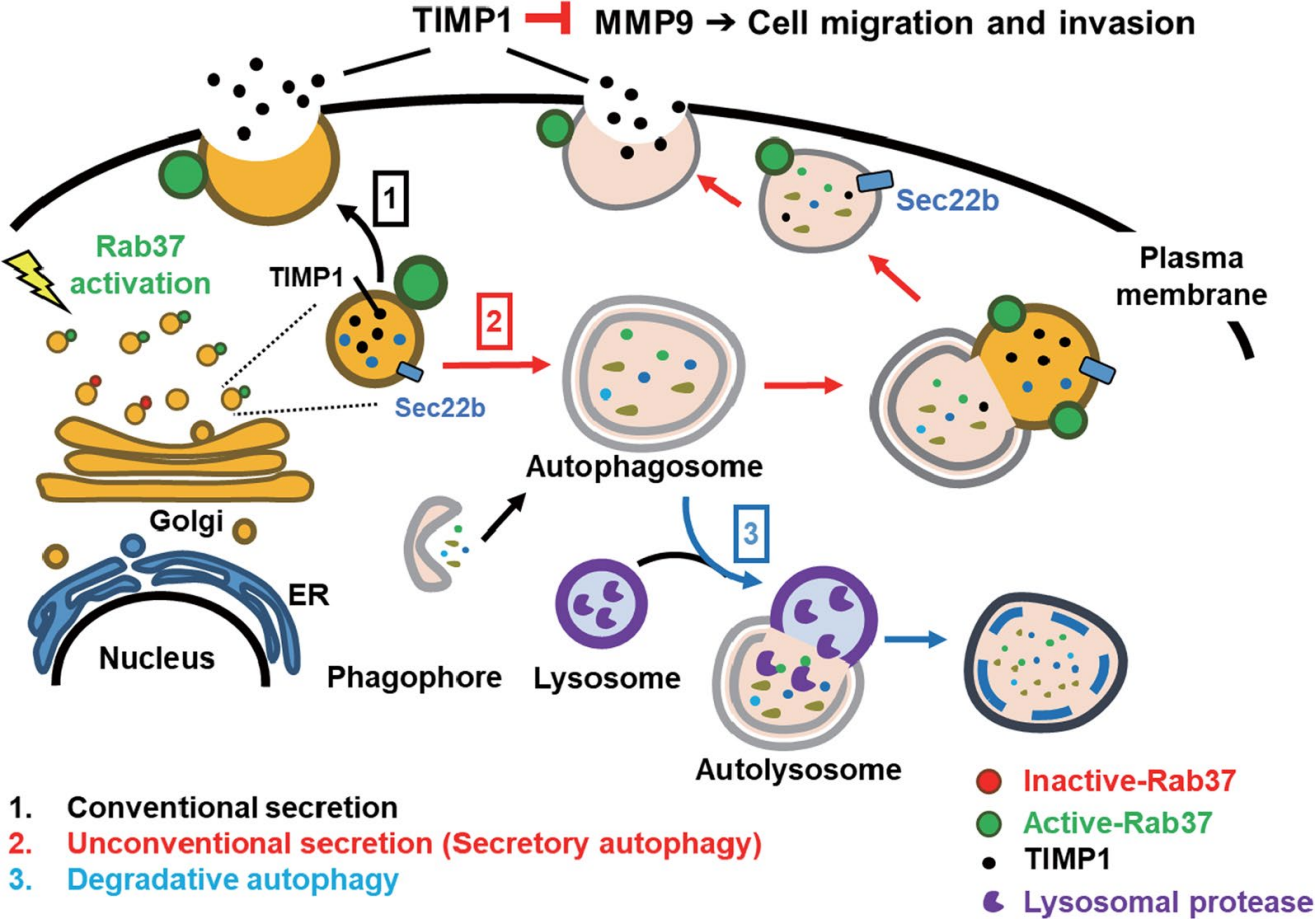
Autophagy promotes active-form Rab37-mediated exocytosis of TIMP1, which suppresses lung cancer cell motility. Autophagy involves degradative and secretory pathways. Rab37 is an upstream regulator of autophagic activity and TIMP1 secretion. Starvation induced active-form Rab37 increases not only TIMP1 secretion but also autophagic activity to promote TIMP1 secretion. Secretory autophagy-mediated TIMP1 secretion is Rab37- and Sec22b-dependent. Secretory autophagy-promoted TIMP1 exocytosis suppresses lung cancer cell motility both in vitro and in vivo

In this study, we investigated the relationship between Rab37 and autophagy as well as the promoting role of autophagy in Rab37-mediated exocytosis of TIMP1 and metastasis both in vitro and in vivo. We found that starvation activated Rab37 (active-form Rab37), and further increased LC3-II through lipidation of LC3-I (unpublished data). Starvation also induced autophagic activity. These two events led to increased LC3-II expression (Fig. 1a). We used two autophagy inducers (amiodarone and niclosamide) and three inhibitors (spautin-1, shRNA of *Atg5* and *Atg7*) to manipulate autophagic activity. The results confirmed that autophagy participates in active-form Rab37-mediated TIMP1 secretion as well as in metastasis of lung cancer cells and the lung-to-lung mouse metastasis model. Autophagy promotes Rab37 to secrete TIMP1 by increasing fusion of the autophagosome with Rab37 and Sec22b-anchored vesicle containing TIMP1 and exocytosis (secretory autophagy) (Fig. 8). Sec22b, a SNARE protein, positively affected the LC3 puncta number and Rab37-LC3 colocalization. Notably, when autophagic activity was induced by amiodarone under Rab37-silencing conditions or under normal medium (Rab37 was not activated), autophagic activity was induced by Tet-D11 (a peptide inducer), but TIMP1 secretion and metastasis could not be induced. It implies that Rab37 is indispensable for autophagy-induced TIMP1 secretion and metastasis (Figs. 2b, d, and 5h–k). The significance of secretory autophagy-related proteins p62 and Sec22b in lung cancer patient specimens as well as in an online big database were analyzed. We revealed that high p62 accumulation (low autophagic activity) was associated with tumor formation and poor overall survival rate. In contrast, no correlation of Sec22b protein with lung cancer patient specimens was detected. This suggests that autophagy, but not membrane-fusing protein Sec22b, plays a crucial role in Rab37-autophagy-mediated TIMP1 secretion and tumorigenesis.

Many laboratories have started to explore non-canonical secretory autophagy. Guo et al. reported that exosome production was decreased in cells lacking the *Atg5* or *Atg16L1* genes, but it was independent of *Atg7* and the traditional autophagic machinery [35]. Atg5 protein specifically decreases the acidification of late endosomes where exosomes are produced and disrupts the acidifying V1V0-ATPase by removing the regulatory component ATP6V1E1 into exosomes. The effect of Atg5 on exosome production promotes the migration and metastasis of orthotropic breast cancer cells. The above findings reveal how the *Atg5* gene regulates exosome release and contributes to metastasis in an autophagy-independent manner. In contrast, we discovered that active-form Rab37 increased autophagic activity and TIMP1 secretion, which led to suppression of lung cancer cell metastasis from the right lung to the left lung (Fig. 5a, b, e and f), as well as a decrease in tumor nodules (Fig. 5c, g). In Fig. 5c, the lack of a statistically significant difference in tumor nodule numbers between the Vector-NT group and the Q89L-NT group was possibly caused by the individual variance in the mice’ genetic background. TIMP1 can influence diverse tumorigenic processes, including proliferation, apoptosis, and metastasis [36–38]. TIMP-1 is known as an inhibitor of MMPs. D'Costa et al. reported that treatment of pancreatic ductal adenocarcinoma (PDAC) with the anticancer drug gemcitabine leads to the upregulation of TIMP1. They demonstrated that TIMP1 played a role in tumor clonogenic survival and vascular density, while TIMP1 inhibition resensitized tumors to gemcitabine and radiotherapy. However, several studies report that high levels of TIMP-1 in the most aggressive tumors, such as colorectal cancer and breast cancer, were associated with poor outcome [39, 40]. In contrast, a high level of TIMP-1 inhibited cancer cell motility, through the inhibition of MMP, leading to breakdown of ECM and basement membranes [41]. The discrepant findings related to the effects of TIMP-1 on tumorigenesis may be explained, at least in part, by the possible MMP-independent functions of TIMP-1, which could facilitate cancer cell progression [42]. TIMP-1 has been proposed as a potential prognostic biomarker in several solid cancers, including breast and colon cancer [43]. In addition to the contradictory role of TIMP1 in tumor growth and metastasis, very little is known about the secretion function of TIMP1. Although gemcitabine-induced TIMP1 secretion attenuates the therapeutic response and promotes tumor growth and liver metastasis in pancreatic cancer [41], the detailed mechanism of TIMP1 secretion regulated by secretory autophagy remains elusive.

Autophagy is known to play dual roles in cancer tumorigenesis [44, 45]. Some studies reported that autophagy is upregulated in response to cancer treatments and protects tumor cells by increasing treatment resistance. Others reported low autophagic activity or autophagy deficiency in various cancers, including hepatoma and colorectal cancer [8, 46]. Nevertheless, these findings imply that pharmacological manipulation of autophagic activity to suppress tumorigenesis is a promising anti-cancer strategy [44, 45, 47–49]. For example, amiodarone, an mTOR inhibitor and an autophagy inducer, could reduce liver injury and promote liver regeneration after partial hepatectomy, and amiodarone-treated patients as well as mice showed no evident toxicity [50, 51]. Lee et al. reported that amiodarone-induced autophagy plays a protective role in the death of lung epithelial cells and pulmonary toxicity [52]. Here, we are the first to reveal that amiodarone treatment could suppress lung cancer metastasis. Furthermore, Wu et al*.* revealed a prophylactic effect on hepatocellular carcinoma occurrence after long-term regular amiodarone usage in a retrospective analysis of case–control data from 32,625 patients in a nationwide, population-based claims database in Taiwan [29]. Niclosamide is a medication used to treat tapeworm infestations and is another mTOR inhibitor and an autophagy inducer [30]. It inhibits the signaling pathways including Wnt/β-catenin, mTORC1, Stat3, NF-κB, and Notch, but also damages tumor cell mitochondria, inhibits proliferation, and induces apoptosis and autophagy. The anticancer effect of niclosamide has been demonstrated both in vitro and in a xenograft mouse model of human colon cancer [53]. Sack et al. showed the suppressive effect of niclosamide on the migration and the invasion of CRC cells overexpressing S100 calcium-binding protein A4 (S100A4), which is essential for metastasis of CRC [54, 55]. These reports showed that diverse pharmacological reagents including amiodarone and niclosamide, had been identified as mTOR inhibitors and autophagic inducers, which are potential candidates as therapeutic agents for the treatment of diverse cancers with low autophagic activity.

Autophagy progression involves several fusion steps which require SNAREs. SNAREs are required to fuse the transport vesicle membrane with an acceptor compartment. At least 36 SNARE members are localized in different membrane compartments in mammalian cells. Sec22b is a SNARE family protein and is involved in vesicle and membrane fusion in secretory autophagy. Under starvation conditions, silencing of Sec22b resulted in decreased levels of LC3-II and LC3 puncta number (Fig. 6). Similar to our finding, Sec22b siRNA suppressed traumatic brain injury (TBI)-induced autophagy in a rat TBI model [56]. Nair et al*.* demonstrated that Sec22b might contribute to the fusion of ATG9 vesicles at the phagophore assembly site [57]. Therefore, Sec22b is involved in the formation of autophagosome [58]. In this study, the roles of Sec22b in Rab37 and secretory autophagy-mediated TIMP1 secretion were clarified by the genetic silencing of Sec22b. Under starvation conditions, silencing of Sec22b expression in lung cancer H460 cells decreased TIMP1 secretion (Fig. 6a). Furthermore, colocalization of LC3 and Rab37 was also decreased in the Sec22b-silenced cells (shSec22b) compared to control groups (Fig. 6b, NT and shGFP). These results indicate that Sec22b participates in TIMP1 secretion, which functionally affects cancer cell mobility (Fig. 8).

Sec22b is one component of the syntaxin-5 SNARE complex, which also contains Bet1, GS27, Sly1, Ykt6, and Sec1–Munc18 (SM) protein [59]. The syntaxin-5 SNARE complex regulates transport in the early conventional secretory pathway between the endoplasmic reticulum (ER) and Golgi apparatus in eukaryotic cells [60]. Furthermore, in the later stage of autophagy, the syntaxin-5 SNARE complex regulates lysosomal protease activity (cathepsins) by controlling anterograde transport from the ER to the Golgi body [61]. Similarly, Renna et al*.* reported that silencing of Sec22b for 48 h also impaired autophagy flux, which leads to increased colocalization of autophagosome and lysosome, and accumulation of autophagosomes in the cytosol in HeLa cells. Depletion of Sec22b impaired the ER-to-Golgi transport in eukaryotic cells [62], which led to the dysfunction of the anterograde transport of lysosomal proteases and accumulation of autophagosomes and decreased degradation of autophagic substrates [61]. Sec22b induced autophagic flux and maturation of lysosomal proteases followed by degradation [63]. In this study, we reveal that knockdown of Sec22b expression decreased TIMP1 secretion together with decreased cell motility, but there was no effect on cell proliferation under starvation conditions.

To clarify whether starvation-induced TIMP1 secretion was caused by secretion but not by cell lysis, we analyzed cell viability by LDH assay under serum starvation conditions. Compared to the positive control cells, all of the cell lines used in this study under starvation treatment for 24 h showed very low LDH values (Additional file 2: Fig. S2, lane 3 to lane 10 vs. lane 1). LDH was slightly increased at 72 h (Additional file 2: Fig. S2, lane 12). This data indicates that the TIMP1 detected in the condition media was due to secretion but not cell lysis.

## Conclusion

Our in vitro and in vivo data imply that secretory autophagy plays a promoting role in active-form Rab37-mediated TIMP1 secretion, which suppresses the motility of lung cancer cells. We are the first to use an off-label drug, amiodarone, to induce autophagy in the lung-to-lung metastasis mice model and to demonstrate that amiodarone-induced secretory autophagy could increase TIMP1 secretion and suppress the motility of lung cancer cells both in vitro and in vivo.

## Supplementary Information

**Additional file 1: Figure S1.** Protein levels in the purified autophagosome and the post-nuclear supernatant from H460 cells. After centrifugation, the whole cell lysate of H460 cells was divided into: (1) the fraction of the post-nuclear supernatant (PNS); (2) the fraction of purified autophagosomes (AP). The levels of TIMP1, Rab37, LC3, and calreticulin (endoplasmic reticulum marker) in PNS and AP fractions were evaluated using specific antibodies by immunoblotting.

**Additional file 2: Figure S2.** The effect of starvation on the viability of the parental H460 and various derivative cell lines at 24, 48, and 72 h. Cells (2 × 10^4^/well) were seeded onto a 96-well plate. H460 parental cells were treated with serum-free DMEM for 24, 48, or 72 h. H460 derivative cell lines: shGFP, shAtg5, shAtg7, shSec22b, VAMP8 KD, shCon, and shRab37 were treated with serum-free DMEM for 24 h. The death of these cell lines was determined by LDH assay.

## Data Availability

The datasets used and/or analyzed in the current study are available from the corresponding author on reasonable request.

## Abbreviations

NSCLC: Non-small-cell lung cancer
SCLC: Small-cell lung cancer
MMP: Matrix metalloproteinases
TIMP1: Tissue inhibitor of metalloproteinase 1
LC3: Microtubule-associated protein 1A/1B-light chain 3
CM: Conditional medium
ATG: Autophagy-related genes
AD: Amiodarone
